# Giant mucinous ovarian cystadenoma mimicking malignancy: a case report and review of diagnostic challenges

**DOI:** 10.1093/jscr/rjag412

**Published:** 2026-05-31

**Authors:** Kağan Gökçe, Murat Üner, Nur Adil, Mehrdad Sheikhvatan

**Affiliations:** Istanbul Okan University, Faculty of Medicine, Department of General Surgery, Surgical Oncology Unit, Istanbul, Turkey; Istanbul Okan University, Faculty of Medicine, Department of General Surgery, Surgical Oncology Unit, Istanbul, Turkey; Istanbul Okan University, Faculty of Medicine, Department of General Surgery, Surgical Oncology Unit, Istanbul, Turkey; Istanbul Okan University, Faculty of Medicine, Department of General Surgery, Surgical Oncology Unit, Istanbul, Turkey

**Keywords:** abdominal mass, adnexial mass, ovarian tumor, mucinous cystadenoma

## Abstract

Benign giant ovarian masses have become less common due to advances in diagnostic and imaging techniques. These tumors require careful diagnosis and surgical management due to their considerable size, despite their benign character. Radiological examinations provide important data regarding lesion size and morphology; however, definitive distinction among neoplasm types is generally achieved only through histopathological evaluation following surgical excision. Therefore, meticulous surgical planning and thorough pathological analysis are essential, especially when managing large adnexal masses. This case report presents a giant adnexal mass originating from the left ovary that underwent surgery and was histopathologically consistent with an ovarian mucinous cystadenoma, along with a review of the current literature.

## Introduction

Adnexal masses are frequently encountered in clinical practice, which range from benign cysts to invasive malignancies. The primary goal of the clinical approach is to manage the symptomatic mass, assess the risk of malignancy, and plan the surgical management. Ultrasonography (US) is the primary imaging modality in the initial evaluation. In large adnexal masses, computed tomography (CT) and magnetic resonance imaging (MRI) provide complementary information regarding the size of the mass, its relationship with adjacent organs, and surgical planning. However, imaging findings and serum tumor markers cannot always provide a definitive distinction between benign, borderline, and malignant lesions, especially in giant and complex mucinous ovarian masses; therefore, the definitive diagnosis is made by histopathological examination after surgical excision in most cases [1, 2].

Mucinous ovarian tumors often present as unilateral, multiloculated cystic lesions and are evaluated within a morphological spectrum consisting of benign mucinous cystadenoma, borderline mucinous tumor, and mucinous carcinoma [2]. Mucinous cystadenomas are among the benign epithelial ovarian neoplasms, but they can sometimes reach enormous sizes and present with symptoms such as abdominal distension, pain, constipation, and other symptoms due to compression of adjacent organs. In reported cases of giant mucinous cystadenoma, it has been emphasized that the size of the mass significantly affects the clinical picture and that surgical treatment is the primary approach for both symptom control and definitive diagnosis [3].

This case report presents a giant adnexal mass originating from the left ovary, measuring 24 × 21 cm that underwent surgery and was histopathologically consistent with an ovarian mucinous cystadenoma, along with a review of the current literature.

## Case report

A 71-year-old female patient, gravida 3, para 3, abortus 0 (G3P3A0), presented with a three-year history of abdominal rigidity on palpation, bloating, and constipation, accompanied by a large intra-abdominal mass. Transvaginal and transabdominal US identified a multicystic, multilobulated mass with dense internal contents, extending to the diaphragm.

Abdominal US demonstrated a multiloculated cystic mass measuring approximately 250 × 150 × 150 mm, extending from the abdominal midline to both upper quadrants. The lesion contained echogenic septa and locules with internal contents of heterogeneous echogenicity. No significant pathological abnormality was detected in other intraabdominal organs.

Given the considerable size of the mass, CT and MRI were employed to assess its characteristics and its anatomical relationship with adjacent organs.

For further lesion characterization, preoperative contrast-enhanced whole-abdomen CT and upper abdominal and pelvic MRI were performed. Contrast-enhanced whole-abdomen CT revealed a multiloculated complex cystic mass measuring approximately 234 × 150 × 180 mm in maximum dimensions, occupying most of the abdominal cavity and extending into the pelvis. The lesion was predominantly hypodense, with focally lobulated contours and enhancing internal septations after intravenous contrast administration.

**Figure 1:**
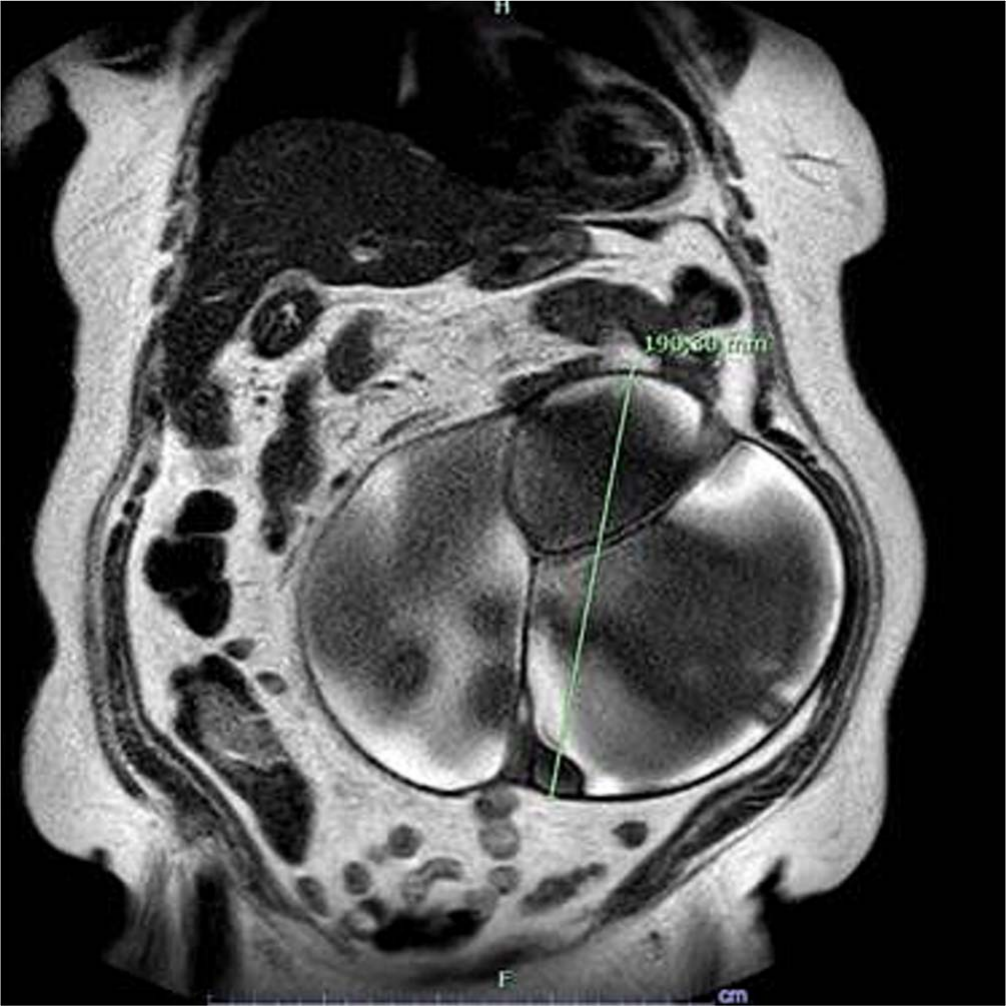
The view of magnetic resonance imaging of the mass (a multiloculated complex cystic mass occupying a large portion of the abdominal cavity and extending inferiorly to the pelvic inlet).

MRI demonstrated a multiloculated complex cystic mass occupying a large portion of the abdominal cavity and extending inferiorly to the pelvic inlet, measuring approximately 24 × 19 × 17 cm (Fig. 1). The lesion contained thin internal septations, which showed enhancement after intravenous contrast administration. Markedly T2-hyperintense areas and scattered foci of T1 hyperintensity were observed within the mass, primarily suggestive of mucinous/proteinaceous content. No definite solid component was identified within the lesion.

Overall, the imaging findings were considered most suggestive of a left adnexal mucinous neoplasm.

**Figure 2:**
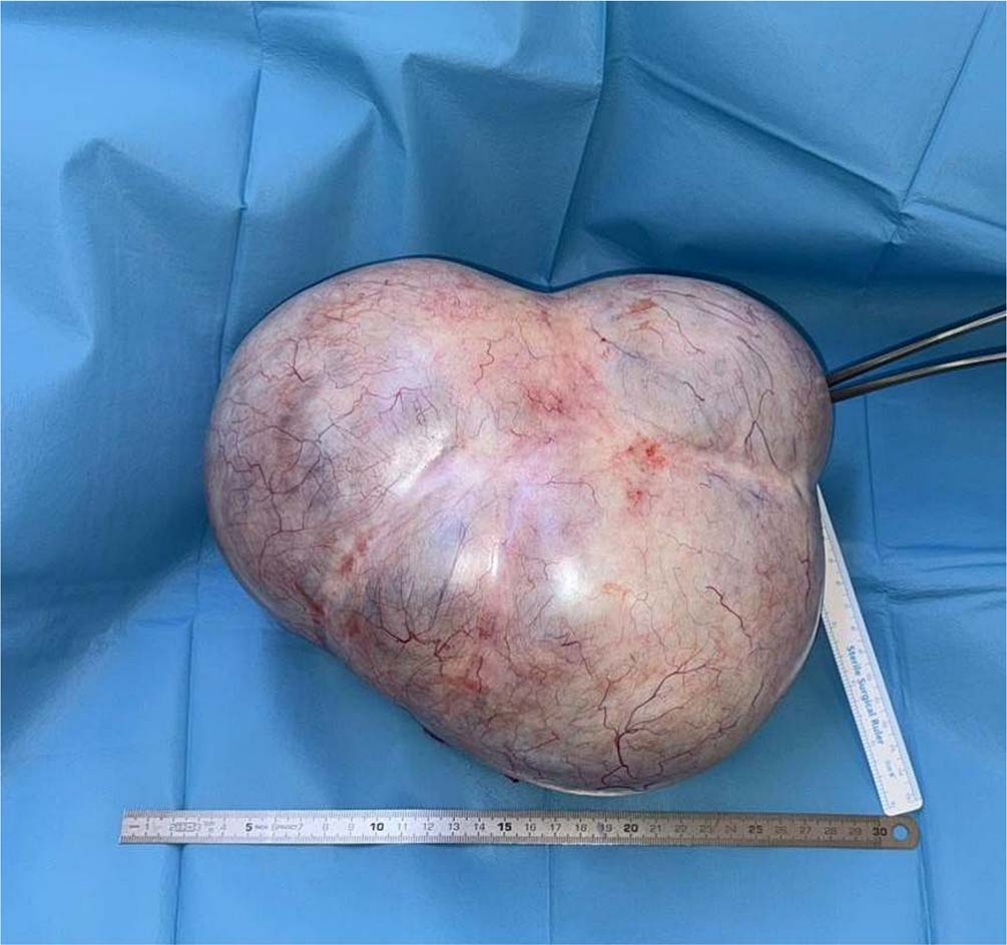
An exploratory laparotomy of the mass (the mass measured 300 × 280 mm, weighed 4200 grams, and exhibited a smooth, lobulated surface).

An exploratory laparotomy was conducted. A midline abdominal incision exposed a mass that completely occupied the abdominal cavity and compressed adjacent organs. Reactive peritoneal fluid was observed, and a sample was collected for cytological examination. The mass measured 300 × 280 mm, weighed 4200 grams, and exhibited a smooth, lobulated surface (Fig. 2). It originated from the left ovary. The mass was carefully separated from the omentum and intestines. The ovarian artery and vein were ligated, and the mass was excised together with the uterine serosa and fallopian tube. Oral feeding commenced 8 hours after surgery. The patient was discharged without complications on the second postoperative day. Histopathological analysis confirmed a mucinous cystadenoma (cytology: reactive mesothelial cells and lymphocytes). The patient’s body mass index (BMI) was calculated before and after surgery. Preoperative BMI was 34, and postoperative BMI was 31.6.

Although preoperative imaging suggested an ovarian origin of the mass, a definitive distinction between benign and malignant mucinous lesions in giant adnexal masses cannot be established preoperatively. In this patient, negative tumor markers and a well-defined cystic lesion on radiological assessment indicated benignity; however, a definitive diagnosis was achieved through histopathological examination following surgical excision.

## Discussion

Giant ovarian mucinous cystadenomas are rare lesions, yet they remain clinically important because, despite their benign histology, their large size may create diagnostic uncertainty and considerable surgical challenges [4, 5]. Radiologically, mucinous ovarian neoplasms typically appear as large, unilateral, multiloculated cystic masses [4, 6]. Variations in mucin content among locules may produce a heterogeneous or classic ‘stained-glass’ appearance on US CT and particularly on MRI [4]. However, imaging modalities do not always allow definitive differentiation between benign mucinous cystadenoma and borderline or malignant mucinous tumors, especially in giant lesions [4–6]. For this reason, features such as mural nodules, papillary projections, thick septa, solid components, bilaterality, ascites, and extraovarian spread should be evaluated with particular caution [4, 6]. In current practice, US is the first-line imaging modality, MRI offers improved characterization of indeterminate adnexal masses, and CT is more useful for evaluating disease extent when malignancy is suspected [7–9]. Although standardized classification systems such as O-RADS have improved preoperative risk stratification, definitive diagnosis still relies on histopathological examination [5, 7–9].

In these cases, the main surgical objective is controlled and complete excision of the mass without intraoperative rupture or spillage of cyst contents [10–13]. Because preoperative imaging cannot entirely exclude borderline or malignant foci, intact removal of the specimen allows more reliable pathological assessment [5, 10]. Although minimally invasive approaches have been reported in selected giant ovarian cysts with predominantly benign clinical and radiological features, these outcomes depend on careful patient selection, a low suspicion of malignancy, and the use of specialized techniques to prevent leakage [10, 11, 13]. In contrast, laparotomy remains the gold standard for very large, multiloculated, or radiologically complex masses, as it provides better exposure, enables more controlled manipulation, and increases the likelihood of intact specimen removal [10, 12]. Moreover, giant ovarian tumors may cause significant perioperative problems, including impaired venous return, restricted diaphragmatic movement, ventilation-related complications, and hemodynamic instability; therefore, multidisciplinary preoperative planning and meticulous intraoperative management are essential [12]. In our case, histopathological confirmation of a 24 × 19 × 17 cm mass as a benign mucinous cystadenoma demonstrates that even very large adnexal masses may still be benign [4, 5]. At the same time, it underscores the importance of careful radiological assessment and meticulous surgical planning to achieve safe resection and accurate diagnosis [4–6, 10–12].

## References

[1] Carvalho JP, Moretti-Marques R, da Silva Filho AL. Adnexal mass: diagnosis and management. RBGO 2020;42:438–43. 10.1055/s-0040-1715547 32736396 PMC10316833

[2] Babaier A, Ghatage P. Mucinous cancer of the ovary: overview and current status. Diagnostics (Basel) 2020;10:52. 10.3390/diagnostics10010052 31963927 PMC7168201

[3] Somagutta MR, Luvsannyam E, Jain MS et al. A rare case of massive ovarian mucinous cystadenoma with postmenopausal bleeding. Cureus 2020;12:338–43. 10.7759/cureus.10198 PMC7532860 33033676

[4] Marko J, Wolfman DJ, Aubin AL et al. Mucinous neoplasms of the ovary: radiologic-pathologic correlation. Radiographics 2019;39:982–97. 10.1148/rg.2019180221 31283462 PMC6677283

[5] Talia KL, Parra-Herran C, Mc Cluggage WG. Ovarian mucinous and seromucinous neoplasms: problematic aspects and modern diagnostic approach. Histopathology 2022;80:255–78. 10.1111/his.14399 33963606

[6] Flicek KT, VanBuren W, Dudiak K et al. Borderline epithelial ovarian tumors: what the radiologist should know. Abdom Radiol (NY) 2021;46:2350–66. 10.1007/s00261-020-02688-z 32860524

[7] Rizzo S, Cozzi A, Dolciami M et al. O-RADS MRI: a systematic review and meta-analysis of diagnostic performance and category-wise malignancy rates. Radiology 2023;307:e220795. 10.1148/radiol.220795 36413127

[8] Zhang Q, Dai X, Li W. Systematic review and meta-analysis of O-RADS ultrasound and O-RADS MRI for risk assessment of ovarian and adnexal lesions. AJR Am J Roentgenol 2023;221:21–33. 10.2214/AJR.22.28396 36722758

[9] Vara J, Manzour N, Chacón E et al. Ovarian adnexal reporting data system (O-RADS) for classifying adnexal masses: a systematic review and meta-analysis. Cancers (Basel) 2022;14:3151. 10.3390/cancers14133151 35804924 PMC9264796

[10] Wang X, Li Y, Wang H. Comparison of perioperative outcomes of single-port laparoscopy, three-port laparoscopy and conventional laparotomy in removing giant ovarian cysts larger than 15 cm. BMC Surg 2021;21:171. 10.1186/s12893-021-01205-3 33882918 PMC8061010

[11] Dubuisson J, Eperon I, Dehaene A et al. Laparoscopic management of giant ovarian cysts using the Alexis laparoscopic system®: a case series. Front Surg 2020;7:24. 10.3389/fsurg.2020.00024 32435653 PMC7218052

[12] Cai S, Dai R, Mi J et al. Perioperative management of a patient with a giant ovarian tumor: a case report. Medicine (Baltimore) 2020;99:e22625. 10.1097/MD.0000000000022625 33031322 PMC7544284

[13] Jiang L, Zhao X, Han Y et al. Giant ovarian cysts treated by single-port laparoscopic surgery: a case series. Front Oncol 2021;11:796330. 10.3389/fonc.2021.796330 34956907 PMC8695676

